# Extraction of maize growth stages in the Sanjiang Plain of China from 2003 to 2022 and their spatio-temporal changes in response to meteorological variables

**DOI:** 10.3389/fpls.2025.1558990

**Published:** 2025-08-19

**Authors:** Huichun Ye, Bingrui Zhang, Shanyu Huang, Chaojia Nie, Peng Wei, Minghao Qin, Hongye Wang

**Affiliations:** ^1^ International Research Center of Big Data for Sustainable Development Goals, Beijing, China; ^2^ Key Laboratory of Digital Earth Science, Aerospace Information Research Institute, Chinese Academy of Sciences, Beijing, China; ^3^ Key Laboratory of Earth Observation of Hainan, Hainan Aerospace Information Research Institute, Sanya, China; ^4^ College of Geoscience and Surveying Engineering, China University of Mining and Technology, Beijing, China; ^5^ Beijing Keda Tongrui Technology Co., Ltd, Beijing, China; ^6^ College of Water Resources Science and Engineering, Taiyuan University of Technology, Taiyuan, China; ^7^ School of Transportation and Geomatics Engineering, Shenyang Jianzhu University, Shenyang, China; ^8^ Cultivated Land Quality Monitoring and Protection Center, Ministry of Agriculture and Rural Affairs, Beijing, China

**Keywords:** Sanjiang Plain, maize, growth stage, agricultural remote sensing, spatio-temporal variation

## Abstract

How to quickly monitor the growth process of maize on a large scale is crucial for regional maize growth assessment, yield estimation, and farmland management. This article takes the Sanjiang Plain in Northeast China as the research area, which is the main grain production area in China. Using MODIS NDVI time series data and Savitzky Golay and Whittaker filtering techniques, a remote sensing extraction method for key growth stages of maize (i.e., jointing stage, tasseling stage, and maturity stage) was established. The spatiotemporal characteristics of these growth stages from 2003 to 2022 were analyzed, alongside their meteorological influences. Results show the Whittaker filter achieves high accuracy, with errors under 8 days. Jointing stages typically fall between June 9^th^ and June 25^th^, tasseling stages between July 20^th^ and August 5^th^, and maturity stages between September 13^th^ and September 29^th^. From 2003 to 2022, jointing and tasseling stages advanced by 0.43 and 0.19 days/year, respectively, while the maturity stage was delayed by 0.38 days/year, indicating an extended growing season correlated with rising surface temperatures and precipitation in the preceding month. These findings offer theoretical and technical guidance for crop growth monitoring, yield assessment, and farmland management.

## 1 Introduction

Black soil, characterized by high fertility and suitability for vegetation growth, is a precious resource endowed by nature. The black soil zones of China serves as a crucial grain production base for the country. Its grain output constitutes approximately one-fourth of the national total, while its commodity quantity accounts for about one-fourth, and the transferred quantity represents around one-third (He et al., 2024). The Sanjiang Plain, a renowned black soil region with a total area of 108,900 ha, was once known as the “Northern famine” but is now referred to as “Beidacang”. It stands as a significant commodity grain base in China, particularly recognized as the production hub of China’s maize commodity grain, one of the world’s most important food crops (Luo C. et al., 2020). Consequently, timely and accurate monitoring of maize crops in the Sanjiang Plain is of paramount significance for advancing the agricultural economy and formulating food policy.

The crop growth stage, also known as phenological period, is a natural biological phenomenon of temporal cycles influenced by anthropogenic activities and environmental factors (Fatima et al., 2020; Xiao et al., 2021; Liu et al., 2021). This period corresponds signifies when crop growth and development attain a critical state. The dynamic changes in regional crop growth stages serve as vital indicators in response to climate and environmental changes. The timely and accurate acquisition of information regarding crop growth stages plays an important role in agricultural monitoring, farmland management, and other related fields. For instance, incorporating growth stage into crop growth monitoring and yield estimation can significantly enhance the accuracy of results (Li et al., 2019; Xie et al., 2021; Luo et al., 2023). Traditional methods for monitoring crop growth stages include manual observation and the accumulated temperature method (Ma and Veroustraete, 2006; Huang et al., 2020). However, these approaches are limited in scope, time-consuming, labor-intensive, and often lack timeliness when monitoring large areas. In contrast, remote sensing measurements can cover extensive regions swiftly, access difficult-to-reach areas, and thus have excellent applicability in the research fields of crop growth monitoring and growth stage feature extraction.

The Normalized Difference Vegetation Index (NDVI) time series data from MODIS can effectively reflects the temporal changes in crops and is widely employed for growth stage and phenological feature extraction (Dunn and de Beurs, 2011; Walker et al., 2014; Cen et al., 2015; Shammi and Meng, 2021; Seguini et al., 2024). However, satellite remote sensing data is influenced by factors such as cloud cover, atmospheric interference and bidirectional reflection. Consequently, NDVI values become saturated when the vegetation coverage is excessively high. Therefore, to utilize NDVI time series data for crop growth stage extraction, it must be de-noised and smoothened (Cai et al., 2017; Li S. et al., 2021; Li X. et al., 2021). Existing studies have demonstrated the efficacy of the Savitzky-Golay (SG) and Whittaker filters in the denoising of time series data (Kandasamy and Fernandes, 2015; Luo Y. et al., 2020). The SG filter exhibits strong fidelity and effectively captures local changes in time series, whereas the Whittaker filter offers a greater balance between fidelity and smoothness, with faster processing speeds (Atzberger and Eilers, 2011; Qiu et al., 2016; Luo et al., 2020; Sáenz et al., 2024). The application and impact of various noise reduction methods on the accuracy of growth stage extraction require further evaluation based on different crop types, terrains, and planting systems (Li et al., 2020).

In the face of global climate change, numerous traditional agricultural practices and crop growth stage characteristics have undergone gradual changes. Crop growth stage serves as a crucial parameter for understanding the crop growth cycle and its response to climate change (Fatima et al., 2020; Xiao et al., 2021; Liu et al., 2021; Yuan et al., 2023; Xu et al., 2024). Studies on the dynamic changes in crop phenology and its underlying mechanisms are instrumental in various applications, including the scientific adjustment and effective management of agricultural production, the development of human responses and adaptation strategies to climate change, and the reduction of agricultural production’s vulnerability and instability (Li et al., 2013; Sisheber et al., 2023). Current research on the spatio-temporal changes of growth stage predominantly focus on vegetation phenology (Piao et al., 2011, 2019; Shao et al., 2021; Gao and Zhao, 2022; Limu et al., 2024), while studies on crop growth stage remain limited. The alterations in the global climate have induced specific changes in crop phenology or crop growth stages (Liu et al., 2017; Wang et al., 2022; Wakatsuki et al., 2023; Rezaei et al., 2023), which consequently impact the management of crop conditions and the formulation of crop production and marketing policies.

This paper focused on the Sanjiang Plain as the research area, utilizing MODIS NDVI time series data and field crop growth stage observation data. It employed Savitzky-Golay and Whittaker filters to fit the time series curves and identify the growth process of maize. The objectives of this study were: (1) to develop remote sensing methods for extracting key growth stages of maize, including the jointing stage, tasseling stage and mature stage, and generate the spatial distribution data products of these stages in the Sanjiang Plain from 2003 to 2022; (2) to analyze the spatiotemporal variation characteristics and meteorological driving factors of the jointing, tasseling and mature stages of maize in the Sanjiang Plain from 2003 to 2022. This study aims to provide important information technology support for maize growth monitoring, yield assessment, and farmland production management in the Sanjiang Plain and similar regions.

## 2 Materials and methods

### 2.1 Study area

**Figure 1:**
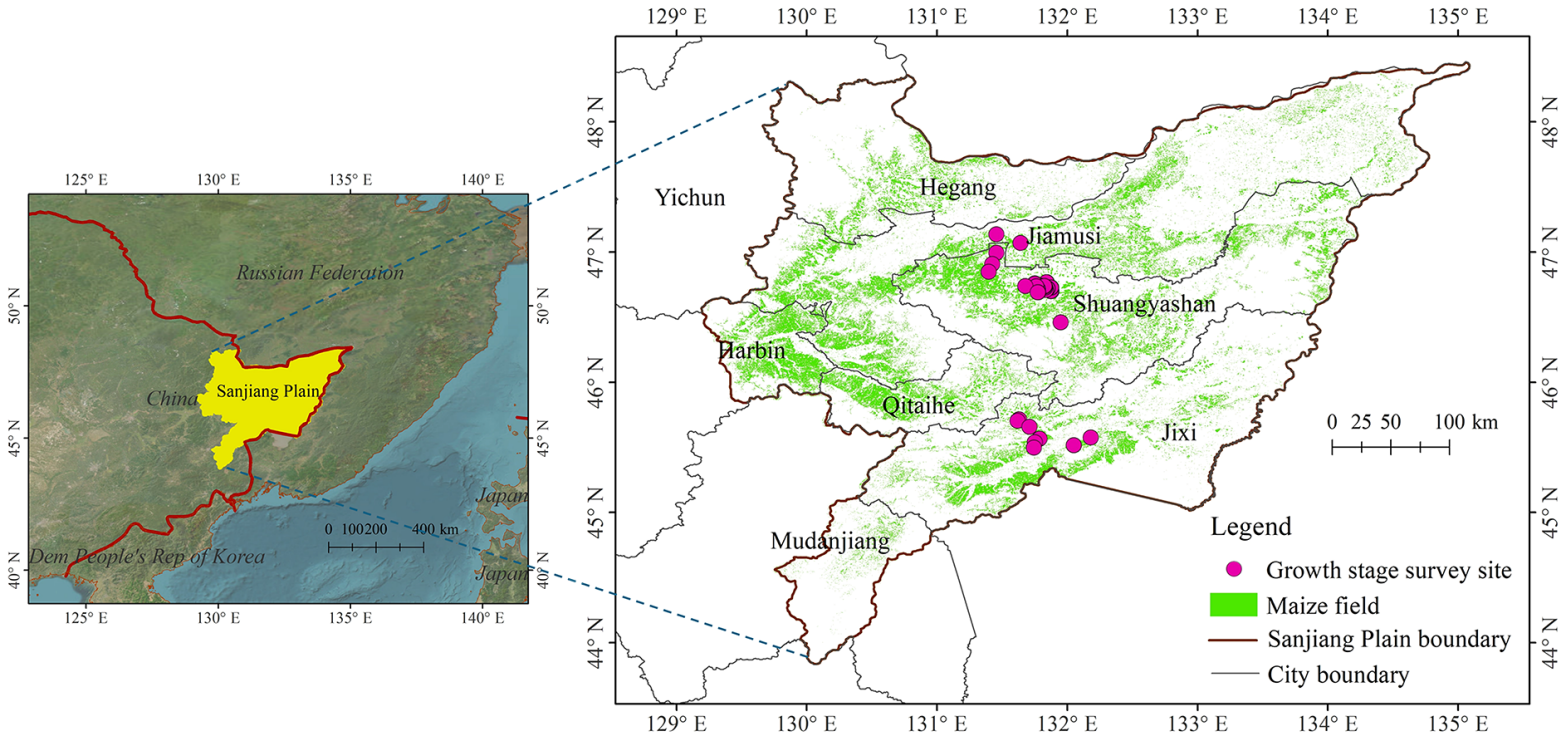
Geographical location of Sanjiang Plain and distribution of survey sites for maize growth stage in this study.

The study area is located in the Sanjiang Plain, which is situated in the northeast of the Northeast Plain in China (**Figure 1**). It is enriched with fertile plain soil formed by the alluvial deposits of the Heilongjiang, Wusuli and Songhua Rivers, spanning approximately 108,900 km^2^. The territory includes 52 farms within the national reclamation system. The climate is defined by a temperate humid and semi-humid continental monsoon type. Annual sunshine hours range from 2,400 to 2,500 hours. The annual temperature changes significantly, with an average temperature of -21 – -18 °C in January and 21 – 22 °C in July. The accumulated temperature above 10°C ranges from 2,300 to 2,500°C. The annual precipitation is 500–650 mm, with 75-85% concentrated from June to October. Rainfall and heat occur in the same season, which is beneficial for agricultural growth. Additionally, the Sanjiang Plain is low and flat, sloping from the southwest to northeast, with an average elevation of 50 ~ 60 m. Maize is a principal crop in the area, cultivated as a single-season annual crop. Most crops are sown in April and reach maturity in September.

### 2.2 Data acquisitions

The datasets used in this study include maize growth stage observations collected during a field campaign, NDVI and surface temperature products derived from MODIS onboard NASA’s Terra and Aqua satellites, the hourly precipitation product from GSMaP Operational, and vector data of the entire Sanjiang Plain.

#### 2.2.1 Field investigation data

Observations of maize growth stage were performed in the Sanjiang Plain during June and July 2022. When a growth period is observed on the plant or stem, it is considered to have entered the given growth stage. The growth phase of a crop population is determined by the percentage of plants (stems) in that stage: onset of development at ≥ 10%; common stage of development at ≥ 50%; and completion of development at ≥ 80%. Finally, a total of 54 maize growth stages were investigated, including both the jointing and tasseling stages./ **Figure 1** shows the distribution of survey sites for maize growth stage in this study.

#### 2.2.2 Remote sensing data

MODIS NDVI products, MOD13Q1 and MYD13Q1, were acquired from the Google Earth Engine (GEE). These datasets comprise 8-day time series with a spatial resolution of 250 meters. In total, 520 data points spanning April to October from 2003 to 2022 were collected. Additionally, the MODIS daily surface temperature product, MYD11A1, was also obtained from GEE, with a spatial resolution of 1000 meters. A total of 200 day and night LST datasets from May to September of 2003–2022 were utilized. Hourly precipitation data from GSMaP Operational, with a spatial resolution of 10 kilometers, were employed. A total of 100 datasets from May to September between 2003 and 2022 were downloaded from GEE.

#### 2.2.3 Maize distribution dataset

Utilizing the 2022 maize distribution dataset in the Sanjiang Plain as the foundational map data, which was obtained using Sentinel-2 time series imagery and the random forest algorithm, with an overall accuracy exceeding 90% (Wei et al., 2023).

### 2.3 Research methods

#### 2.3.1 NDVI time series filtering

The products derived from satellite remote sensing data often contain artefacts induced by cloud cover, precipitation, and various other environmental impediments. Notably, the NDVI time-series data is prone to erratic and significant fluctuations, which necessitates the application of smoothing filters. This investigation employs two filtering techniques: the Savitzky-Golay filter and the Whittaker filter.

##### 2.3.1.1 Savitzky-Golay filter

The Savitzky-Golay filter, introduced by Savitzky and Golay (1964), is a method of least-squares convolution fitting designed for the smoothing and derivative calculation of contiguous or spectral data points. It constitutes a form of weighted moving average filter, wherein the weights are determined by the polynomial order employed in the least-squares fitting within the filter’s window. This polynomial is crafted to retain significant data values while mitigating the impact of outliers. Applicable to any continuous and moderately smooth dataset with uniform intervals, the filter is particularly suitable for NDVI time series data. The process of smoothing NDVI time series data via the least-squares convolution method can be articulated as shown in Equation 1.


(1) $$Y_{j}^{'}=\frac{\sum_{i=-m}^{i=m}C_{i}Y_{j+i}}{N}$$

where *Y* is the original NDVI value; $Y^{\prime }\;$ is the fitting value; $C_{i}$ is the filtering coefficient of the *i*-th NDVI value; N is the number of convolutions, which is also equal to the size of the smoothing window  (2m+1 ); and j refers to the *i-*th data in the NDVI time series. The smooth array contains (2m+1 ) points and m is half the size of the smoothing window.

##### 2.3.1.2 Whittaker filter

The Whittaker filter operates on the principle of compensated least squares. This filtering algorithm adeptly harmonizes the fidelity and smoothness of time series data, automatically yielding optimally filtered curves. Additionally, it is computationally efficient and maintains signal integrity during the smoothing phase, as indicated in reference (Atzberger and Eilers, 2011). The underlying concept is as follows: Given a sequence y, of length N, uniformly sampled, the goal is to derive a smooth sequence z from y by striking a balance between two competing objectives: data integrity and the smoothness of z. A smoother *z* inherently sacrifices some data fidelity, as it diverges from *y* actual values, and vice versa. The fitting efficacy, denoted as Q, can be articulated as shown in Equations 2-4.


(2) Q=S+λR

(3) $$S=\sum_{i}{\left(y_{i}-z_{i}\right)}^{2}$$

(4) $$R=\sum_{i}{\left(z_{i}-3z_{i-1}+3z_{i-2}-z_{i-3}\right)}^{2}$$

where *S* is the fidelity, *R* is the roughness, and *λ* is the user parameter. The goal of the algorithm is to determine the sequence z that minimizes *Q*. The larger the value of *λ*, the greater the impact of *R* on the objective function *Q*, and the smoother *z* becomes, and vice versa. In this study, the λ value is adjusted by minimizing the error between the smoothed data and the observed data, iteratively seeking the λ that results in the smallest error. This ensures that the smoothed data not only removes noise but also retains critical crop phenological characteristics.

#### 2.3.2 Cubic spline interpolation

Cubic spline interpolation is widely used in time-series spectral curve analysis. Its key advantages lie in low computational cost, high fidelity, and consistent continuity, making it ideally suited for applications where smooth interpolation between data nodes is critical (Cai et al., 2017; Li et al., 2021). By constructing a set of third-degree polynomials, the method ensures the interpolated curve is smooth at each data location and in its vicinity, with continuous first and second derivatives. As a result, interpolated spectral index series can exhibit more continuous and precise trends (Qin et al., 2024).

In this study, the resampled NDVI time series data, initially captured at an 8-day interval, was interpolated to a daily resolution using cubic spline interpolation (Vorobiova and Chernov, 2017), thereby aligning with the critical phenological stages of crop growth expressed in Julian days.

#### 2.3.3 Crop growth stage extraction

**Figure 2:**
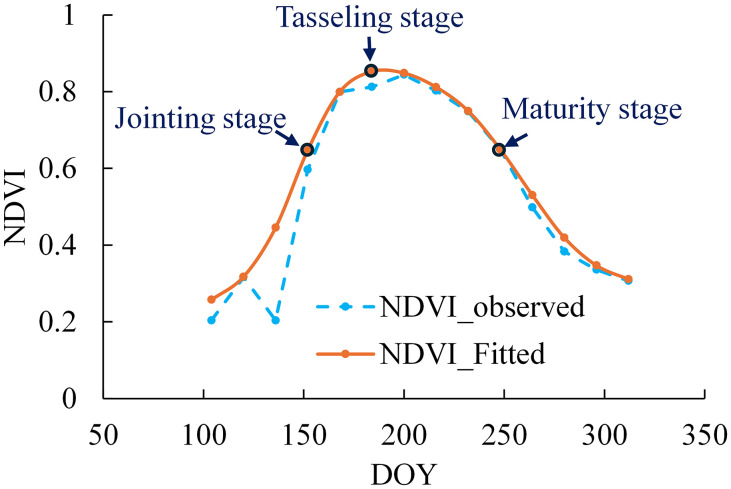
Schematic diagram of maize growth stage extraction based on NDVI time series filtering method.

Based on the temporal characteristic curve of spectral indices, a curve that reflects the crop’s growth trajectory, the slope feature node method enables the identification of multiple key nodes. The inflection node where the curve exhibits its maximum positive slope corresponds to the period of most rapid vegetative growth, marking the maize jointing stage (Qin et al., 2024). The subsequent peak of the curve, characterized by a zero slope (first derivative = 0), represents the maximum biomass accumulation and signals the transition from vegetative to reproductive growth, coinciding with the tasseling stage (Sakamoto et al., 2005). Furthermore, the point of maximum negative slope in the declining phase indicates the onset of senescence and physiological maturity, corresponding to the harvest-ready stage (Xu and Zhang, 2012) the schematic diagram of maize growth stage extraction based on NDVI time series filtering method as shown in **Figure 2**.

In this study, we systematically identify three critical phenological stages in maize development by analyzing the characteristics of the temporal growth curve: (1) the jointing stage (maximum positive slope, indicating peak growth rate), (2) the tasseling stage (slope = 0, representing maximum biomass), and (3) the maturity stage (maximum negative slope, reflecting rapid senescence).

#### 2.3.4 Research analysis and verification methods

##### 2.3.4.1 Person correlation analysis and trend analysis methods

Person correlation analysis was employed to analyze the relationship between various maize growth stage datasets and meteorological variables in the Sanjiang Plain from 2003 to 2022. Furthermore, Trend analysis was used to analyze the overall change trend of the growth stages. The correlation coefficient was used as the accuracy evaluation index, and its calculation formula is shown in Equation 5:


(5) $$R_{xy}=\frac{\sum_{i=1}^{n}[(x_{i}-\bar{x})(y_{i}-\bar{y})]}{\sqrt{\sum_{i=1}^{n}{\left(x_{i}-\bar{x}\right)}^{2}\sum_{i=1}^{n}{\left(y_{i}-\bar{y}\right)}^{2}}}$$

where $R_{xy}$ is the correlation coefficient between various growth stages and meteorological variables, such as surface temperature or precipitation. If $R_{xy}$ surpasses the threshold of statistical significance in the t-test at the 0.01 or 0.05 level, it denotes an extremely significant or significant trend, respectively; otherwise, there is no significant relationship. n refers to the number of years from 2003 to 2022 (n=20 in this study), $x_{i}$ is the meteorological parameter data for the *i*-th year, and $\bar{x}$ is the average of meteorological parameter (surface temperature or precipitation) data over the n years. $y_{i}$ refers to the day of the year (DOY) corresponding to the specific growth stage of maize for the *i*-th year, $\bar{y}$ refers to the average DOY corresponding to the specific growth stage of maize over the n years.

The trend slope was calculated according to Equation 6.


(6) $$b=\frac{\sum_{i=1}^{n}[(x_{i}-\bar{x})(y_{i}-\bar{y})]}{\sum_{i=1}^{n}{\left(x_{i}-\bar{x}\right)}^{2}}$$

where b is the slope, indicating the magnitude of the change trend. If b <0, it indicates that the specific growth stage of maize has advanced; otherwise, it indicates that the growth stage has been delayed.

##### 2.3.4.2 Evaluation indicators

In this study, the performance of maize growth stage extraction was evaluated using three metrics: the root mean square error (RMSE), the mean absolute error (MAE) and the mean absolute percentage error (MAPE). The corresponding formulas are as as shown in Equations 7-9.


(7) $$RMSE=\sqrt{\frac{1}{n}\sum_{i=1}^{n}{\left(Y_{i}-Y_{i}^{'}\right)}^{2}}$$

(8) $$MAE=\frac{1}{n}\sum_{i=1}^{n}|Y_{i}-Y_{i}^{'}|$$

(9) $$MAPE=\frac{1}{n}\sum_{i=1}^{n}|\frac{Y_{i}-Y_{i}^{'}}{Y_{i}}|\times 100\%$$

where $Y_{i}$ is the filtered NDVI value or the maize growth period extracted based on remote sensing image; $Y_{i}^{'}$ refers to the growth period of maize spot in Sanjiang Plain based on original NDVI value or field experiment. *n* is the number of samples. Low MAE, RMSE, and MAPE values indicate high model prediction accuracy (Ruan et al., 2025).

## 3 Results and analysis

### 3.1 Verification of extraction accuracy of maize growth stage using various filtering methods

**Figure 3:**
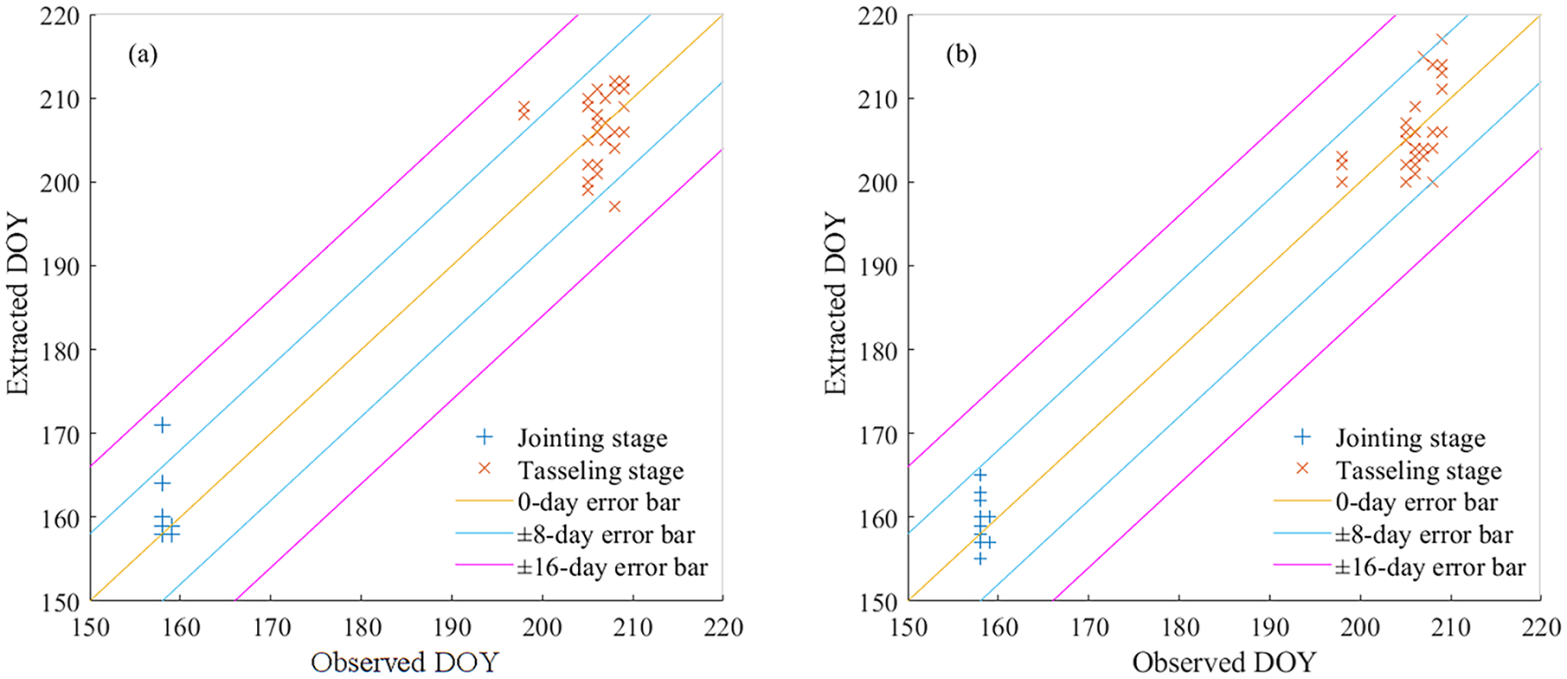
Comparison of observed and extracted day of year (DOY) values for the maize growth stage using various filtering methods in the Sanjiang Plain in 2022. **(a)** Savitzky-Golay filter, and **(b)** Whittaker filter.

The day of year (DOY) values for maize jointing and tasseling stages in 2022 were extracted using Savitzky-Golay and Whittaker filtering approaches. The extraction accuracy of these methods was validated by comparing against ground-based observational data from 2022. Validation against ground-based phenological observations revealed the superior performance of Whittaker filtering (**Table 1**), as evidenced by consistently lower error metrics (RMSE, MAE, and MAPE) for both growth stages compared to Savitzky-Golay filtering. Furthermore, as illustrated in **Figure 3**, the discrepancies in the Whittaker filter-extracted dates for these stages were within the ± 8-day error bar, whereas the Savitzky-Golay filter yielded errors that occasionally surpassed this threshold, albeit remaining within ±16-day error bar. These findings suggest a superior performance of the Whittaker filter in the accurate delineation of maize growth stages. Consequently, for the ensuing analyses in this investigation, the Whittaker filter was selected as the preferred method for the extraction and analysis of maize growth stage data.

**Table 1:** The validation results of observed and extracted day of year (DOY) values for the maize growth stage using various filtering methods in the Sanjiang Plain in 2022.

Growth stage	Savitzky-Golay filter			Whittaker filter		
	RMSE	MAE	MAPE	RMSE	MAE	MAPE
Jointing Stage (day)	4.77	2.84	1.80	3.58	2.68	1.70
Tasseling Stage (day)	5.29	4.11	2.01	4.34	3.83	1.85

### 3.2 Temporal and spatial variation of maize growth stages in the Sanjiang Plain

#### 3.2.1 Characteristics of the spatial distribution of maize growth stages

**Figure 4:**
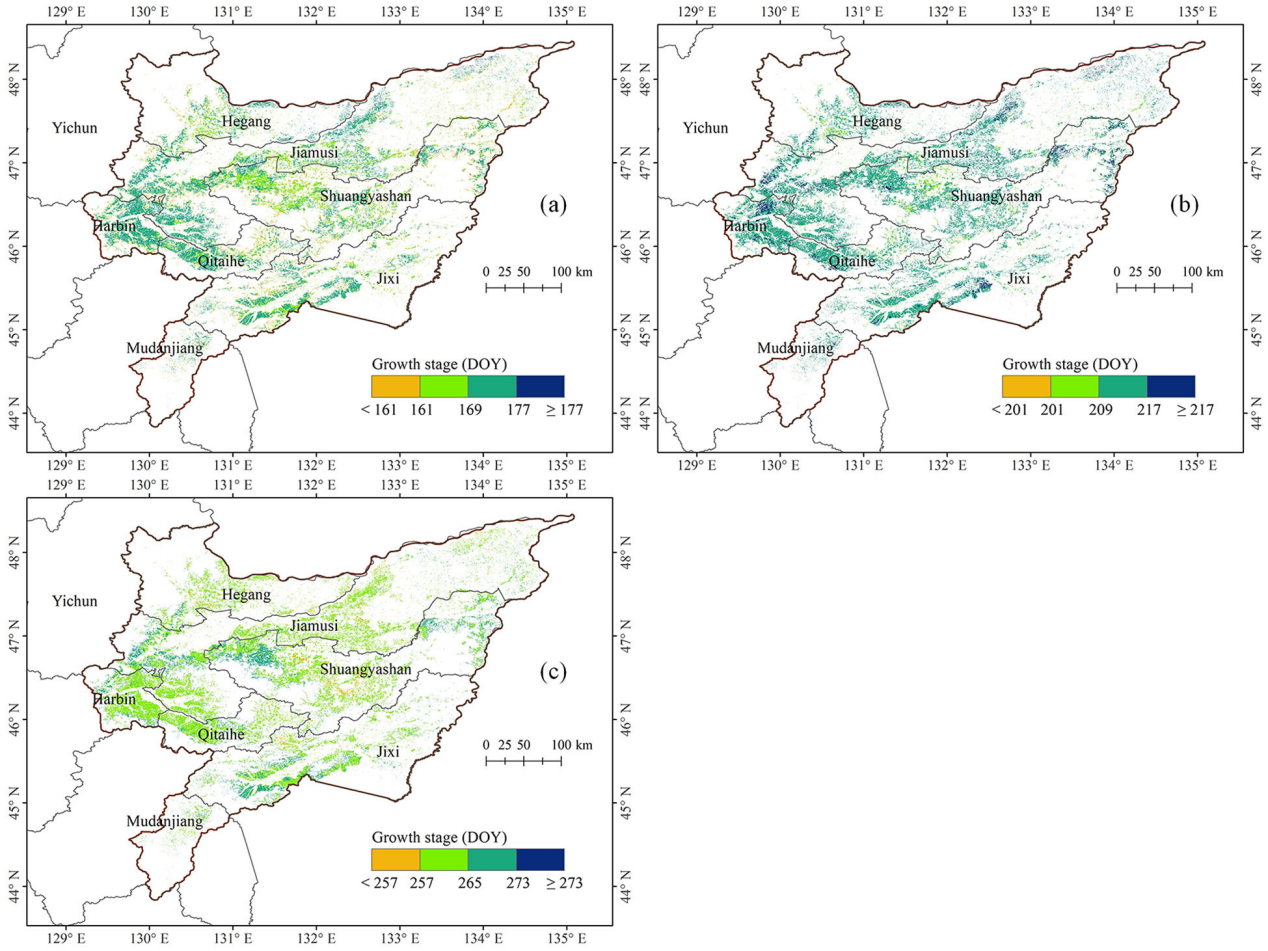
Spatial distribution of maize growth stages in the Sanjiang Plain in 2022. **(a)** Jointing stage, **(b)** tasseling stage, and **(c)** maturity stage.

**Figure 4** presents the spatial distribution characteristics of key maize growth stages in 2022 across the Sanjiang Plain. The maize’s jointing stage was largely concentrated around DOY 161 and 177, spanning from June 9th to June 25th, with an average occurrence at DOY 168, corresponding to June 17th. An overwhelming majority of 85.21% of the study area was in this phase. Approximately 14.30% of the jointing stage occurred before June 9th, whereas 0.49% occurred after June 25th. In terms of spatial distribution, the jointing stage of Hegang City in the northwest and Shuangyashan City in the central region occurred from June 9th to June 17th; In contrast, the outbreaks in Harbin City, Qitaihe City, Jixi City, and Jimusi City occurred from June 17th to June 25th.

The tasseling stage of maize in the Sanjiang Plain predominantly occurred within the span of DOY 201 to 217, specifically from July 20th to August 5th, with an average of DOY 213, corresponding to June 31st. This period accounted for 90.61% of the study area. A mere 0.84% of the tasseling events transpired prior to July 20th, while 8.56% occurred subsequent to August 5th. The peak tasseling stage in the Sanjiang Plain was typically observed from July 28th to August 5th.

The maturity stage of maize was predominantly concentrated between DOY 257 and 273, ranging from September 13 to September 29, with an average at DOY 263, corresponding to September 19. This period accounted for 96.82% of the study area. Roughly 3.18% of the Sanjiang Plain’s maize reached maturity before September 13th. In the western regions of Jiamusi City and Shuangyashan City, as well as the southwestern sector of Jixi City within the Sanjiang Plain, a scattered few maize fields matured between September 21st and September 29th. Elsewhere, the bulk of maize maturity was observed between September 13th and September 21st.

#### 3.2.2 Characteristics of interannual variability in maize growth stages

**Figure 5:**
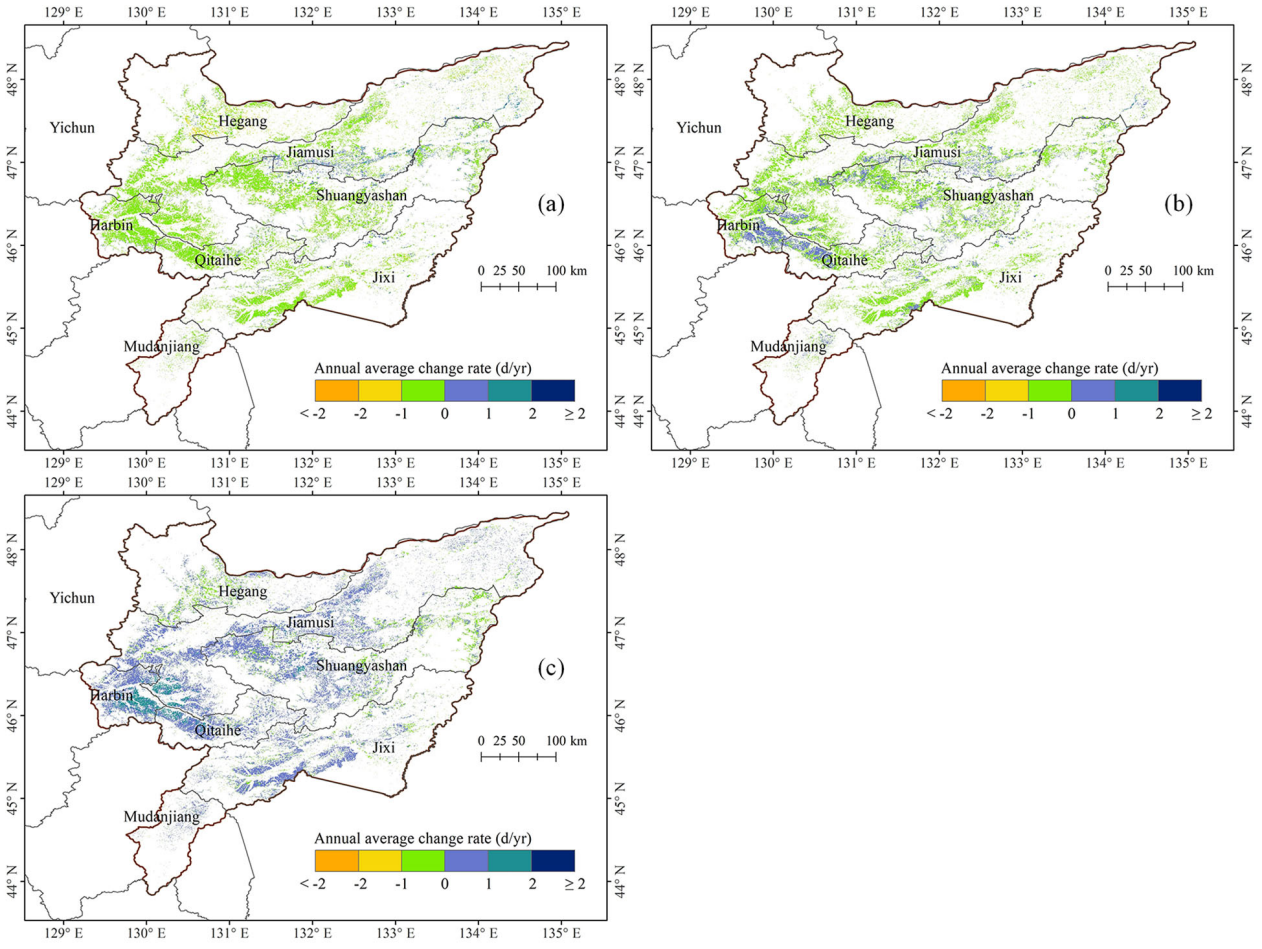
Spatial distribution of annual average change rate of maize growth stages in the Sanjiang Plain from 2003 to 2022. **(a)** Jointing stage, **(b)** tasseling stage, and **(c)** maturity stage.

**Figure 5** illustrates the interannual trends in the growth stages of maize cultivation across the Sanjiang Plain from 2003 to 2022. There was a notable propensity for the maize’s jointing phase to commence earlier, with an average advancement rate of 0.43 days per annum encompassing 86.90% of the region, whereas only 13.0% demonstrated a tendency towards delay. Notably, the jointing stage in Hegang City, Jixi City, and the northeastern part of Harbin City has all advanced, with 5.21% of the areas advancing by an average of more than one day per year (-1 d/yr). In contrast, a significant delay in the jointing stage was identified in the central and northeastern sectors of Jiamusi City, and the northeastern areas of Qitaihe and Shuangyashan Cities, with 1.52% showing a delay surpassing one day per year (+1 d/yr).

The tasseling stage exhibited a general trend of advancing, with an average of -0.19 d/yr, covering 75.90% of the total area of the study area. In contrast, the region experiencing a delayed tasseling phase constitutes a mere 24.10%. Notably, the northern and western sectors of Hegang City, Jiamusi City, and the majority of Jixi City witnessed a marked forward shift in the tasseling phase, with approximately 0.66% of the territory demonstrating an advancement exceeding one day per year (-1 d/yr). Conversely, certain areas in the northeast of Harbin, the western part of Qitaihe City, the northwest of Shuangyashan City, and the southern part of Jixi City showed a significant delay in tasseling stage, with about 0.23% of the areas delayed by more than one day (+1 d/yr).

The maturity stage showed a delayed trend, with an average delay of 0.38 d/yr, covering 81.47% of the study area. Conversely, only 18.53% of the regions indicated an advancement trend. Specifically, small areas such as the central area of Hegang City, the eastern part of Shuangyashan City, and the northeastern part of Jixi City have significantly advanced maturity, with 0.04% of the areas experiencing an advance of more than one day (-1 d/yr). However, significant maturity delay was observed in the most areas of Jimusi City and Qitaihe City, the northeastern part of Harbin City, and western part of Shuangyashan City, with 6.35% of the regions experiencing a delay of more than one day (+1 d/yr).

### 3.3 The influence of meteorological factors on the maize growth stages in the Sanjiang Plain

#### 3.3.1 The influence of meteorological factors on the maize growth stages

**Figure 6:**
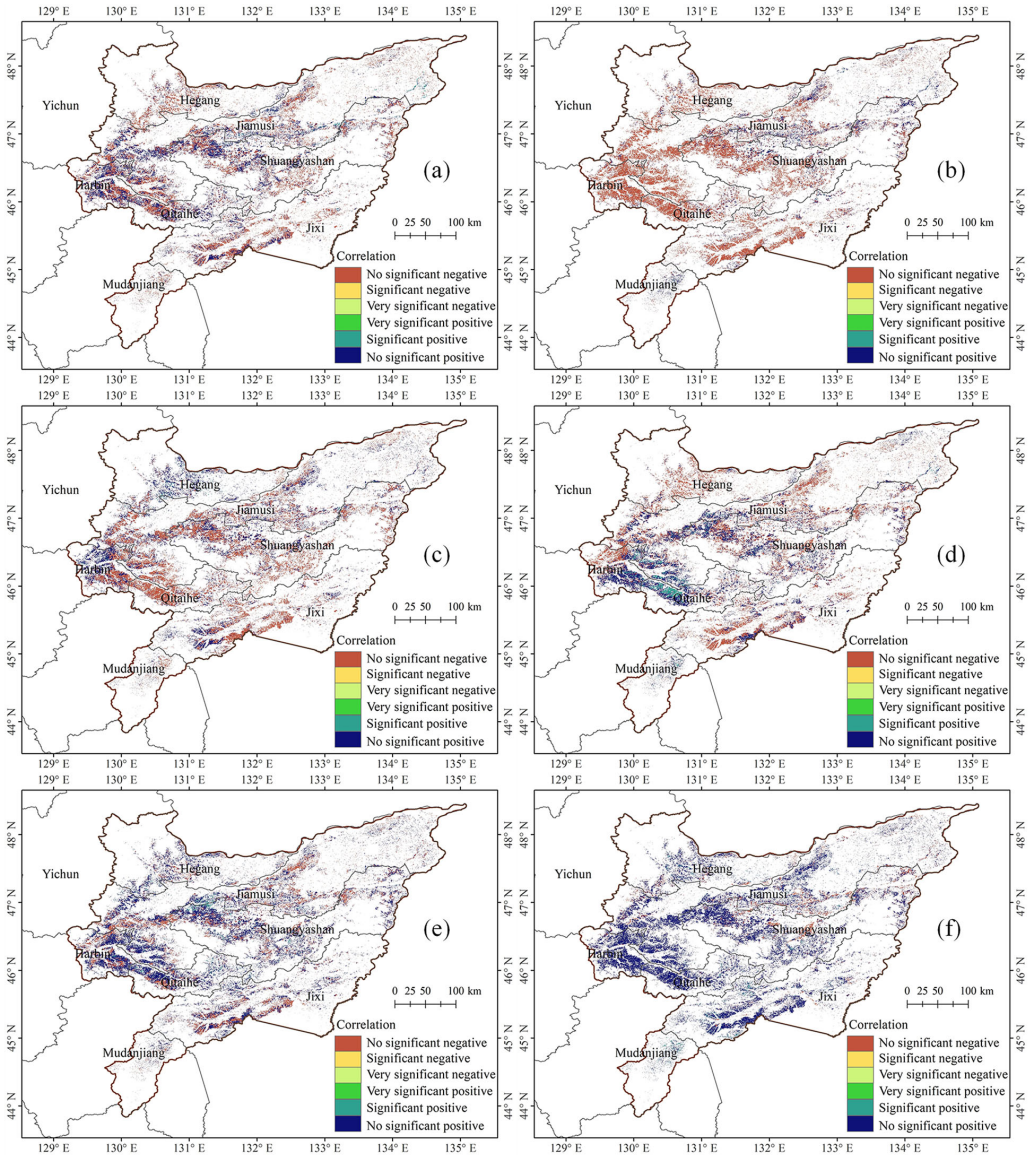
Distribution map of correlation between the growth stages of maize and the meteorological factor in Sanjiang Plain from 2003 to 2022. **(a)** Jointing stage & average land surface temperature in May, **(b)** jointing stage & accumulated precipitation in May, **(c)** tasseling stage & average land surface temperature in June, **(d)** tasseling stage & accumulated precipitation in June, **(e)** maturity stage & average land surface temperature in August, and **(f)** maturity stage & accumulated precipitation in August.

This study used correlation analysis to quantitatively investigate the relationship between the growth stages of maize and the meteorological factors (including monthly average temperature and monthly cumulative precipitation) in the Sanjiang Plain from 2003 to 2022. The findings show that the jointing, tasseling, and maturation stages of maize occur in June, July, and September, respectively. Considering the delayed impact of climatic conditions on maize growth, this study analyzed meteorological data from the month preceding these growth stages. T-tests were employed to determine the significance of the correlation coefficients: p < 0.01 indicated an extremely significant correlation; p < 0.05 indicated a significant correlation; and p ≥ 0.05 indicated a non-significant correlation. **Figure 6** shows the distribution map of correlation between the growth stages of maize and the meteorological factor in Sanjiang Plain from 2003 to 2022.

The results indicate that there is spatial heterogeneity in the correlation between different growth stages of maize and the meteorological factors of the preceding month, characterized by the coexistence of positive and negative correlation areas. For the jointing stage, the correlation between its DOY value and the average land surface temperature in May shows a negative correlation in 58.57% of the areas and a positive correlation in 41.43% of the areas (**Figure 6a**). Regarding the correlation with the accumulated precipitation in May, it is negative in 80.10% of the areas and positive in only 19.90% of the areas (**Figure 6b**). For the tasseling stage, the correlation between its DOY value and the average land surface temperature in June shows a negative correlation in 60.21% of the areas and a positive correlation in 39.79% of the areas (**Figure 6c**). As for the correlation with the accumulated precipitation in June, it is negative in 54.77% of the areas and positive in 45.23% of the areas (**Figure 6d**). For the maturity stage, a different trend is observed. The correlation between its DOY value and the average temperature in August is positive in 64.94% of the areas and negative in 35.06% of the areas (**Figure 6e**). The correlation with the total precipitation in August is even more pronounced, being positive in as high as 83.74% of the areas and negative in only 16.26% of the areas (**Figure 6f**). However, the T-test results indicate that, regardless of temperature or precipitation, the correlation is relatively weak in the vast majority of areas. This suggests that meteorological factors at the monthly scale have limited explanatory power for maize phenological variation, implying that we need to consider the cumulative effects of meteorological factors and the coupling interactions among multiple factors.

#### 3.3.2 The maximum meteorological influencing factor on maize at different growth stages

**Figure 7:**
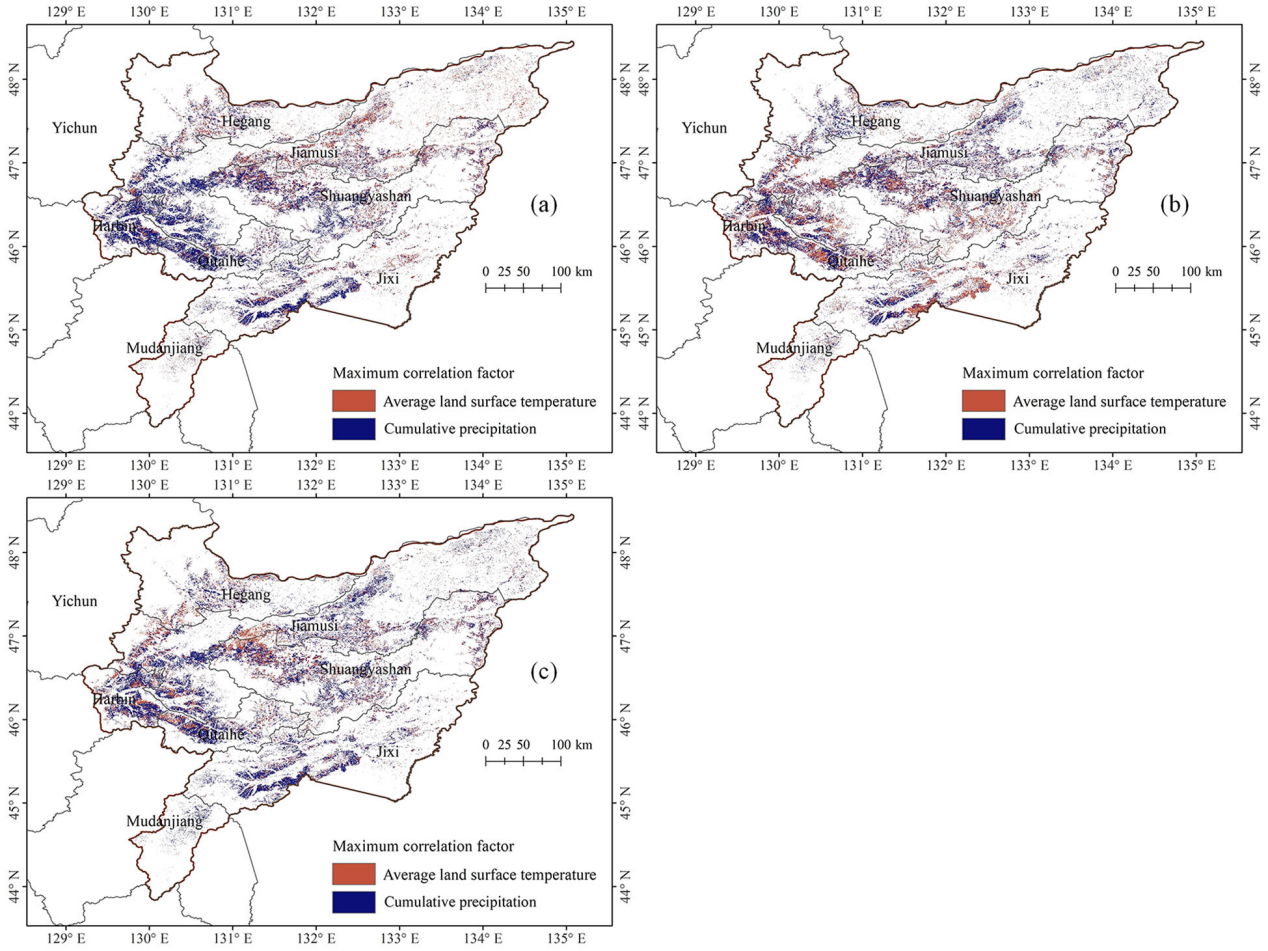
Distribution map of maximum meteorological influencing factors on maize at different growth stages in the Sanjiang Plain from 2003 to 2022. **(a)** Jointing stage, **(b)** tasseling stage, and **(c)** maturity stage.

Regional climatic variations lead to differing effects of temperature and precipitation on maize growth, suggesting that the primary meteorological factors influencing maize cultivation vary by location. This study further investigated the maximum meteorological related factors of maize growth stages in different regions of the Sanjiang Plain, as shown in **Figure 7**. During the jointing stage, 63.33% of the area was more influenced by precipitation than by average land surface temperature in May, primarily in the southwest of the Sanjiang Plain; conversely, 36.67% of the area, mainly in the northeast, was more affected by average land surface temperature than by precipitation. For the tasseling stage, 54.21% of the area was more impacted by June precipitation than by June average land surface temperature, scattered across various regions of the Sanjiang Plain; while 45.79% of the area, located in the central and southern parts, was more influenced by average land surface temperature than by precipitation. In the maturity stage, 64.42% of the area was predominantly affected by August precipitation over August average land surface temperature, mainly in the southern and southwestern regions; whereas 35.58% of the area, scattered in the central and northeastern parts, was more impacted by average land surface temperature than by precipitation.

## 4 Discussion

In this study, two methods—the Savitzky-Golay (SG) filter and the Whittaker filter—were employed to fit the MODIS NDVI time series curve. The findings revealed that the curves fitted by these methods closely resembled the original ones, maintaining high fidelity, in alignment with previous studies (Atzberger and Eilers, 2011; Luo et al., 2020; Sáenz et al., 2024). These time series curves were instrumental in extracting the growth stage characteristics of maize in the Sanjiang Plain and were validated using ground measurement data. The results indicated that both filtering methods demonstrated commendable extraction accuracy, with the Whittaker filter outperforming the SG filter in terms of performance. The growth stage error derived from the Whittaker method’s fitted curve is typically within 8 days, whereas the error for the SG filtering method is within 16 days, though predominantly within 8 days. This may be attributed to the Whittaker filter’s ability to more flexibly control signal smoothing by adjusting the smoothing parameter while effectively reducing boundary distortion, making it particularly effective in handling non-linear or non-periodic signals (Kong et al., 2019; Khanal et al., 2020). Additionally, the analysis errors arise from the impact of clouds, aerosols, and other atmospheric factors on remote sensing satellite data, as the noise from these elements cannot be entirely eliminated through filtering methods. Furthermore, while MODIS data offer the advantage of long-term continuity, this study employs a synthesized data product with an 8-day temporal resolution and a 250-meter spatial resolution. To achieve a 1-day temporal resolution, interpolation was performed, which may introduce some errors. The spatial resolution of MODIS data being 250 meters means that maize planting areas contain mixed pixels, further influencing the extraction of growth stage results. In the future, higher-resolution satellite data (i.e., Sentinel-2) could be considered for such studies to meet the needs of more refined monitoring and management.

This study demonstrates that there are significant spatial and temporal variations in the growth period of maize in the Sanjiang Plain. The significant changes in climate conditions, especially key factors such as temperature and precipitation, across different years and regions profoundly affect the growth and development process of maize. This study systematically analyzed the key growth stage trends of maize in the Sanjiang Plain from 2003 to 2022 and their correlations with average surface temperature and cumulative precipitation, providing a scientific basis for developing agricultural adaptation strategies in the context of future climate change. The findings revealed an overall trend of advancement in the maize jointing stage, while the tasseling and maturity stages exhibited a general trend of delay. These results align closely with the findings of Li et al. (2011). The jointing stage typically shows a negative correlation with surface temperature and precipitation; the tasseling stage also generally shows a negative correlation with these factors, whereas the maturity stage exhibits a positive correlation with surface temperature and precipitation. Against the backdrop of global climate change, maize in the Sanjiang Plain from 2003 to 2022 demonstrated an overall trend toward earlier planting and later harvesting, along with a lengthened growing season, which is consistent with previous studies (Li et al., 2011, 2013; Zhang et al., 2017). Furthermore, evaluating the robustness of this method over long time series is crucial for enhancing the robustness of the proposed technology. In future work, it is necessary to carry out the collection of multi-year verification-related data to comprehensively evaluate the performance and robustness of the method over long time spans.

While this study has made significant progress in characterizing changes in key growth stages of maize in the Sanjiang Plain over the past two decades, several limitations remain. First, the validation for this study utilized only 54 ground samples; the limited sample size may affect the reliability of the evaluation and the model’s universality. Furthermore, since the validation was based on data from a single year (2022), it limits a comprehensive assessment of the method’s performance over the entire 2003–2022 period. Additionally, the assumption that the maize planting distribution remained unchanged for 20 years (based solely on 2022 data), while in reality there were local variations, further reduced the accuracy of the results. Second, analytically, this study solely employed correlation coefficient analysis to examine the relationship between maize phenological stages and meteorological factors. Although correlation analysis is a commonly used tool for assessing relationships, it may be insufficient for fully revealing the potential nonlinear interaction mechanisms between crop phenology and complex climatic conditions. Furthermore, the results indicate that in most cases, no significant correlation was found between the occurrence timing of maize growth stages and corresponding meteorological factors (monthly mean temperature and cumulative precipitation) from the preceding month. This phenomenon likely stems from multiple intertwined factors. For instance, this study only considered meteorological data from one month prior to each growth stage, whereas crop development is typically a cumulative process more closely related to accumulated temperature and precipitation from sowing onwards. Additionally, crop growth stages themselves represent complex ecological processes influenced by various environmental factors (e.g., temperature, precipitation, solar radiation, relative humidity) (Wang et al., 2019; Sun et al., 2022). Concurrently, anthropogenic factors cannot be overlooked, as farming decisions and management practices including cultivar selection, sowing date, fertilization and irrigation significantly impact the specific timing and spatial manifestation of growth stages (Ayoola and Makinde, 2009; Liu et al., 2010; Lee et al., 2022; Ma et al., 2024). Moreover, spatial heterogeneity in soil types, topography and microclimatic conditions within the region also plays important roles (Ashiq et al., 2021; Chen et al., 2021; Pérez-Hernández et al., 2021; Shuqin et al., 2024), while extreme weather events like droughts and floods may introduce unpredictable constraints (Venkatappa et al., 2021; Furtak and Wolińska, 2023; Iqbal et al., 2025). However, systematically collecting detailed data encompassing all these factors across large regions presents substantial challenges. Therefore, future research should focus on enhancing the collection and integration of relevant multi-source data, while employing more advanced modeling approaches to thoroughly analyze the relative contributions of various factors to spatiotemporal variability in maize phenology, with the goal of achieving a more comprehensive and profound understanding of its driving mechanisms.

This study successfully implemented precise monitoring of crop growth stages, providing farmers with crucial data support and decision-making guidance for aspects such as variety selection, water and fertilizer management, pest and disease control, and field management. The observed advancement of the maize jointing and tasseling stages, coupled with a delayed maturity phase, represents a significant trend in maize phenology under the context of climate change. Of course, factors such as crop variety improvement have also contributed to these changes. A deep understanding of these shifts in growth stages and their underlying driving factors is key to optimizing crop management strategies, effectively addressing the challenges posed by climate change, and ensuring the stability of maize yield and quality. Additionally, monitoring the crop growth period can help farmers accurately assess crop maturity and determine the optimal harvest timing, thereby maximizing the final yield and quality of maize. Moreover, the findings of this research are not only applicable to the current study area and crop types but also provide technical method references for extracting the growth periods of different crops in other geographical regions with varying climatic conditions. With the continuous advancement of machine learning and deep learning technologies, remote sensing extraction of crop growth stages is moving towards more precise and intelligent directions (Adhinata et al., 2024; Ahmed et al., 2024; Naseer et al., 2024). The application of full time-series data, combined with deep learning models, can more comprehensively capture subtle changes in the plant growth process, improving the accuracy and reliability of phenology identification (Chen et al., 2025). The rapid development of AI-driven agricultural monitoring systems will also greatly enhance the timeliness and accuracy of crop phenology monitoring, as well as agricultural decision-making capabilities (Yu et al., 2024, 2025), thereby promoting the development of precision agriculture and smart agriculture.

## 5 Conclusions

This study focuses on the Sanjiang Plain as the research area, utilizing MODIS NDVI time series data along with Savitzky-Golay and Whittaker filtering techniques to establish a remote sensing extraction method for the key growth stages of maize (including the jointing stage, tasseling stage, and maturity stage). Based on this method, we extracted and analyzed the spatiotemporal variation characteristics of maize jointing, tasseling, and maturity stages in the Sanjiang Plain from 2003 to 2022, as well as their influence by meteorological factors. The results indicate that the maze growth stages extracted using the Whittaker filter demonstrate higher accuracy, with errors within 8 days. The extracted growth stages of maize show that the jointing stage was mainly concentrated between DOY 161(June 9th) and DOY 177 (June 25th), the tasseling stage between DOY 201 (July 20th) and DOY 217 (August 5th), and the maturity stage between DOY 257 (September 13) and DOY 273 (September 29). From 2003 to 2022, the jointing and tasseling stages of Maize in the Sanjiang Plain exhibited an advancing trend, with advancements of 0.43 days per year and 0.19 days per year, respectively, while the maturity stage showed a delaying trend, with a delay of 0.38 days per year. This phenomenon is generally reflected in an extended growth season, which is positively correlated with rising surface temperatures and increased precipitation in the month preceding the growth stage. However, the study further indicated that the explanatory power of meteorological factors at the monthly scale (i.e., temperature and precipitation) alone remains limited for variations in maize phenology. This suggests that we need to consider the cumulative effects of meteorological factors as well as the coupling interactions between multiple factors. The research findings provide important information technology support for maize growth monitoring, yield assessment, and farmland production management in the Sanjiang Plain and similar regions.

## Data Availability

The original contributions presented in the study are included in the article/supplementary material. Further inquiries can be directed to the corresponding author.
